# Dynamics of Ionic Liquids in Confinement by Means of NMR Relaxometry—EMIM-FSI in a Silica Matrix as an Example

**DOI:** 10.3390/ma13194351

**Published:** 2020-09-30

**Authors:** Danuta Kruk, Milosz Wojciechowski, Malgorzata Florek-Wojciechowska, Rajendra Kumar Singh

**Affiliations:** 1 Faculty of Mathematics and Computer Science, University of Warmia&Mazury in Olsztyn, Słoneczna 54, 10-710 Olsztyn, Poland; wojciechowski@matman.uwm.edu.pl; 2 Department of Physics & Biophysics, University of Warmia and Mazury in Olsztyn, Oczapowskiego 4, 10-719 Olsztyn, Poland; mfw@matman.uwm.edu.pl; 3 Ionic Liquid and Solid State Ionics Laboratory, Department of Physics, Banaras Hindu University, Varanasi 221 005, India; rajendrasingh.bhu@gmail.com

**Keywords:** ionic liquids, dynamics, diffusion, nuclear magnetic resonance, relaxation

## Abstract

^1^H and ^19^F spin–lattice relaxation studies for 1-ethyl-3-methylimidazolium bis(fluorosulfonyl)imide in bulk and mesoporous MCM-41 silica matrix confinement were performed under varying temperatures in a broad range of magnetic fields, corresponding to ^1^H resonance frequency from 5Hz to 30MHz.A thorough analysis of the relaxation data revealed a three-dimensional translation diffusion of the ions in the bulk liquid and two-dimensional diffusion in the vicinity of the confining walls in the confinement. Parameters describing the translation dynamics were determined and compared. The rotational motion of both kinds of ions in the confinement was described by two correlation times that might be attributed to anisotropic reorientation of these species.

## 1. Introduction

The dynamical properties of ionic liquids in confinement are of high interest from the point of view of fundamental as well as applied science. In both cases, the underlying question concerns the influence of the interactions with the confining walls on the translational and rotational diffusion of the ions. From the perspective of exploiting ionic liquids as safe and efficient electrolytes, the effort is directed towards preserving their fast translational motion (and hence, high conductivity) despite the confinement applied for safety purposes. Independently of the main motivation, the possibilities to enquire into the mechanisms of ionic motion in confinement are very limited. The most “natural” attempt to gain this type of information is to measure the translation diffusion coefficient using nuclear magnetic resonance (NMR) gradient methods. This concept is based on differences in the resonance frequencies of NMR active nuclei (nuclei possessing a spin) caused by the diffusion in a magnetic field with controlled inhomogeneity (a magnetic field gradient) [1,2]. In case of ionic liquids composed of ^1^H containing cations and ^19^F containing ions, one can straightforwardly measure the translation diffusion coefficients of both ions. However, the values of the diffusion coefficients do not provide much information about the mechanism of motion: slow translation diffusion implies low conductivity, but the reason of the slow diffusion remains unknown. One can also perform NMR relaxation studies—the relaxation rates are linked to the time scale of the dynamical processes modulating the spin interactions that cause the relaxation process. This concept sounds attractive because one is approaching the ionic dynamics on the atomistic level by probing the time scale of the fluctuations of spin interactions. “Classical” NMR relaxation experiments are, however, performed at a single, high magnetic field (resonance frequency). According to the spin relaxation theory [3,4,5,6,7], the molecular (ionic) motion occurring on a time scale being of the order of the reciprocal resonance frequency is most efficient as the source of the relaxation process. Consequently, at high resonance frequencies, one probes fast dynamics—in most cases, rotational motion, sometimes combined with fast, short-range translation dynamics. This type of study often causes confusion and disappointment: the observed, fast dynamics does not lead to high conductivity, because of its local character—the long-range translation diffusion being, in fact, responsible for the conductivity, is slow. NMR relaxometry based on the Fast Field Cycling technology [8,9,10,11] allows to perform relaxation experiments in a broad range of magnetic fields, corresponding to the range of about 5 KHz–40 MHz of resonance frequencies (referring to ^1^H nuclei). This implies that one is able to probe, in a single experiment, dynamical processes on the time scales from ms tons [10,12,13]. Moreover, the shape of the relaxation dispersion profile (spin–lattice relaxation rate versus the resonance frequency) unambiguously reveals the mechanism of motion [14,15,16,17], also allowing to differentiate between the translation diffusion pathways of different dimensionality: 3D, 2D, 1D [18,19,20,21,22,23,24,25,26,27]. Relaxation rates are given as linear combinations of spectral density functions being Fourier transforms of the corresponding time correlation functions, characterising the motion associated with the relaxation process [3,4,5,6,7]. The mathematical forms of the correlation functions depend on the mechanism of motion. Consequently, after applying Fourier transform in order to switch from the time-domain to the frequency-domain, the resulted spectral density function (and hence, the shape of the relaxation dispersion profile) becomes a fingerprint of the nature of the dynamical process. In this work, we exploit this unique potential of NMR relaxometry to enquire into the mechanism of ionic motion of 1-ethyl-3-methylimidazolium bis(fluorosulfonyl)imide (EMIM-FSI) in silica confinement compared to the liquid in bulk. A detailed understanding of the ionic dynamics and the identification of the origin of the slowing down of ionic dynamics in confinement are necessary for designing ionogel systems that exhibit high conductivity comparable to liquids in bulk. Moreover, the specifics of molecular and ionic motion in confinement are one of the fundamental subjects of science. In this context, the work can also be considered as an example of how the NMR relaxometry reveals the dimensionality of translation diffusion in confinement. 

The paper is organized as follows. In Section 2, the theoretical models of ^1^H and ^19^F spin–lattice relaxation in bulk and confinement are presented, Section 3 includes experimental details, in Section 4 the experimental data are shown and analysed, in Section 5 the obtained results are discussed, while Section 6 includes the concluding remarks.

## 2. Theory

The primary source of the ^1^H and ^19^F relaxation processes is magnetic dipole–dipole interactions. These interactions can be of inter-molecular (inter-ionic) and intra-molecular (intra-ionic) origin. The inter-molecular interactions fluctuate in time as a result of translation dynamics, while the intra-molecular dipole–dipole couplings are mediated by the rotational dynamics of the molecule (ion). Consequently, the spin–lattice relaxation rate, $R_{1I}$, (*I* denotes ^1^H or ^19^F) is given as a sum of two contributions, $R_{1I}^{trans}$ and $R_{1I}^{rot}$, associated with the translation and rotation dynamics, respectively:

(1) $$R_{1I}\left(\omega_{I}\right)=R_{1I}^{trans}\left(\omega_{I}\right)+R_{1I}^{rot}\left(\omega_{I}\right)$$

where $\omega_{I}$ denotes the resonance frequency (in angular frequency units) of the nucleus. The explicit forms of the relaxation contributions depend on the mechanism of motion. For three-dimensional (3D) translation diffusion, the characteristic of liquids in bulk, the relaxation rate $R_{1I}^{trans}\left(\omega_{I}\right)$ can be expressed as [14,15,16,19,20]:

(2) $$R_{1I}^{trans}\left(\omega_{I}\right)=\frac{108}{5}{\left(\frac{\mu_{0}}{4\pi }\gamma_{I}^{2}\hbar \right)}^{2}\frac{1}{d_{I}^{3}}N_{I}\int_{0}^{\infty }\frac{u^{4}}{81+9u^{2}-2u^{4}+u^{6}}\left[\frac{\tau_{trans}}{u^{4}+{\left(\omega_{I}\tau_{trans}\right)}^{2}}+\frac{4\tau_{trans}}{u^{4}+{\left(2\omega_{I}\tau_{trans}\right)}^{2}}\right]du$$

The correlation time $\tau_{trans}$ is defined as: $\tau_{trans}$ = $\frac{d_{I}^{2}}{2D_{trans}}$, where $d_{I}$ denotes the distance of the closest approach between the molecules (ions) carrying the *I*-nuclei, while $D_{trans}$ denotes their translation diffusion coefficient; $\gamma_{I}$ denotes the gyromagnetic factor of the *I*-nucleus, while $N_{I}$ is the number of the *I*-nuclei (^1^H or ^19^F in this case) per unit volume. The expression of Equation (2) can be expanded into the Taylor series in the limit $\omega_{I}\tau_{trans}\ll 1$ (i.e., in the low frequency range), leading to the relationship [23,24,25]:

(3) $$R_{1I}^{trans}\left(\omega_{I}\right)=R_{1I}^{trans}\left(0\right)-\frac{\sqrt{2}\pi }{15}\left(1+4\sqrt{2}\right){\left(\frac{\mu_{0}}{4\pi }\gamma_{I}^{2}\hbar \right)}^{2}N_{I}{\left(2D\right)}_{}^{-3/2}\sqrt{\omega_{I}}$$

The expression not only allows to straightforwardly determine the translation diffusion coefficient from the low frequency slope of the relaxation rate versus the squared root of the resonance frequency [23], but also enables to unambiguously identify the mechanism of the translation diffusion—the linear dependence of $R_{1I}^{trans}\left(\omega_{I}\right)$ on $\sqrt{\omega_{I}}$ is a fingerprint of the 3D character of the translation motion [19,20,23,24,25,28,29]. In the case of two-dimensional (2D) diffusion, expected for liquids in confinement in the vicinity of the confining walls, the form of the corresponding spectral density changes, leading to the expression [21,24]:

(4) $$R_{1I}^{trans}\left(\omega_{I}\right)=C_{DD,I}^{trans}\tau_{trans}\left[ln\left(1+\frac{1}{{\left(\omega_{I}\tau_{trans}\right)}^{2}}\right)+4ln\left(1+\frac{1}{{\left(2\omega_{I}\tau_{trans}\right)}^{2}}\right)\right]$$

where $C_{DD,I}^{trans}$ denotes a dipolar relaxation constant associated with the translation dynamics. In the low frequency range, $\omega_{I}\tau_{trans}\ll 1$, Equation (4) can be approximated as

(5) $$R_{1I}^{trans}\left(\omega_{I}\right)\propto \left[-\tau_{trans}ln\left(\omega_{I}\tau_{trans}\right)\right]$$

indicating a linear dependence of the relaxation rate on $ln\left(\omega_{I}\right)$ [21,24].

In the simplest case of isotropic molecular (ionic) rotation, an exponential correlation function and hence, a Lorentzian spectral density function, are assumed. Consequently, the relaxation rate takes the form [4,5,6,7]:

(6) $$R_{1I}^{rot}\left(\omega_{I}\right)=C_{DD,I}^{rot}\left[\frac{\tau_{rot}}{1+{\left(\omega_{I}\tau_{rot}\right)}^{2}}+\frac{4\tau_{rot}}{1+{\left(2\omega_{I}\tau_{rot}\right)}^{2}}\right]$$

where $\tau_{rot}$ denotes the rotational correlation time, while $C_{DD,I}^{rot}$ is referred to as a dipolar relaxation constant associated with the rotational dynamics. Due to the fast rotation of the anions in bulk, the relaxation contribution associated with the rotational dynamics turned out to be negligible. On the basis of the outlined models, the ^1^H and ^19^F spin–lattice relaxation process of the EMIM-FSI in bulk and in the confinement can be reproduced in terms of the following expressions:

(7) $$R_{1I}^{bulk}\left(\omega_{I}\right)=\;\frac{108}{5}{\left(\frac{\mu_{0}}{4\pi }\gamma_{I}^{2}\hbar \right)}^{2}\frac{1}{d_{I}^{3}}N_{I}\int_{0}^{\infty }\frac{u^{4}}{81+9u^{2}-2u^{4}+u^{6}}\left[\frac{\tau_{trans,I}^{bulk}}{u^{4}+{\left(\omega_{I}\tau_{trans,I}^{bulk}\right)}^{2}}+\frac{4\tau_{trans,I}^{bulk}}{u^{4}+{\left(2\omega_{I}\tau_{trans,I}^{bulk}\right)}^{2}}\right]du$$

where $R_{1I}^{bulk}\left(\omega_{I}\right)$ denotes the overall relaxation rate for the system in bulk, $\tau_{trans,I}^{bulk}$ and $\tau_{rot,I}^{bulk}$ are the translational and rotational correlation times, respectively ($\tau_{trans,I}^{bulk}$ = $\frac{d_{I}^{2}}{2D_{trans,I}^{bulk}}$, where $D_{trans}^{bulk}$ denotes the translation diffusion coefficient in bulk), while $C_{DD,I}^{rot,bulk}$ denotes the rotational dipolar relaxation constant for bulk:

(8) $$\begin{array}{c} R_{1I}^{conf}\left(\omega_{I}\right)=R_{1I}^{trans,\;conf}\left(\omega_{I}\right)+R_{1I}^{rot,conf,s}\left(\omega_{I}\right)+R_{1I}^{rot,conf,f}\left(\omega_{I}\right)+A_{I}\\ =C_{DD,I}^{trans,conf}\tau_{trans}^{conf}\left[ln\left(1+\frac{1}{{\left(\omega_{I}\tau_{trans}^{conf}\right)}^{2}}\right)+4ln\left(1+\frac{1}{{\left(2\omega_{I}\tau_{trans}^{conf}\right)}^{2}}\right)\right]+\\ C_{DD,I}^{rot,conf,s}\left[\frac{\tau_{rot,I}^{conf,s}}{1+{\left(\omega_{I}\tau_{rot,I}^{conf,s}\right)}^{2}}+\frac{4\tau_{rot,I}^{conf,s}}{1+{\left(2\omega_{I}\tau_{rot,I}^{conf,s}\right)}^{2}}\right]+\;C_{DD,I}^{rot,conf,f}\left[\frac{\tau_{rot,I}^{conf,f}}{1+{\left(\omega_{I}\tau_{rot,I}^{conf,f}\right)}^{2}}+\frac{4\tau_{rot,I}^{conf,f}}{1+{\left(2\omega_{I}\tau_{rot,I}^{conf,f}\right)}^{2}}\right]+A_{I} \end{array}$$

Equation (8) requires more detailed explanations. First, anticipating the results, the translation diffusion of both cations and anions in the confinement is of 2D character—therefore, the expression of Equation (4) was exploited. As far as the rotational dynamics is concerned, it turned out that a single term of this type does not allow to reach a sufficient agreement with the experimental data. Therefore, we used two terms including two sets of parameters with the indexes “*s*” and “*f*”, referring to slow and fast dynamics, respectively. We shall discuss the physical meaning of this approach in the forthcoming sections. The two sets of parameters, $C_{DD,I}^{rot,\;conf,s\left(f\right)}$, $\tau_{rot,s\left(f\right)}^{conf}$ refer to the dipolar relaxation constants in the confinement and the rotational correlation times. Eventually, Equation (8) includes a frequency independent term, *A_I_*. The frequency independent relaxation contribution is associated with dynamical processes being too fast (the correlation time is too short) to lead to a visible dependence of the corresponding relaxation contribution on the resonance frequency. It might originate from the internal dynamics of the confined ions.

## 3. Experimental Details

The ^1^H and ^19^F spin–lattice relaxation measurements were performedusing a commercial relaxometer (Stelarsrl, Spinmaster 2000, Mede, Italy). The magnetization values were measured for 16 linearly spaced time sets, the span of which was readjusted at every relaxation field to optimize the sampling of the decay/recovery curves. Free induction decays were recorded at 16.3 MHz after a single π/2 pulse. For magnetic fields below 11 MHz, pre-polarization at 25 MHz was applied. Temperature was stabilized with an air flux system with the accuracy of 0.5 K.

The ionic liquid, EMIM-FSI (purity ~99.9%) and the ordered mesoporous MCM-41 silica matrix were purchased from Solvionic, Toulous, France and Sigma-Aldrich, Bangalore, India, respectively. Methanol (purity ~99.8%, Sigma-Aldrich) was used as the solvent. Samples were heated at 60 °C in vacuum (10^−3^ torr). All the samples were handled in an Ar filled glove box (Mbraun). The confined system was prepared following the vacuum-assisted physical imbibition process described in detail in [30,31]. The nitrogen-sorption measurement was performed for the determination of the pore parameters and this sample showed a type IV isotherm, characteristic of mesoporous nature [31]. The confined system was prepared following the procedure described in [30,31]. MCM-41 has a mesoporous structure with cylindrical pores and the measured average pore diameter in it is of about 3.5 nm. For the ionic liquid concentration of 70%, the average pore diameter increased to 21 nm [31]. The morphology of the sample was analysed by SEM, TEM and N_2_-sorption measurements. They showed smooth, ordered mesoporous texture with the uniform cylindrical nature of pores. It was also noticed that ion-pairs come closer to each other and behave as a compressed media inside the pores. It was found that as the concentration of the ionic liquid increased, the average pore diameter of the matrix becomes larger. This happens due to the complete filling of small pores of MCM-41. Due to the high capillary suction of active interaction sites present on the pore wall surface of MCM-41, the ionic diffusion is enhanced into empty smaller pore space [31]. The sample was handled in an Ar filled glove box, and hence the moisture (water) content was less than 20 ppm as measured by Karl–Fischer titrator., Mettler-Toledo India Private Limited, Mumbai, India.

## 4. Results and Analysis

**Figure 1:**
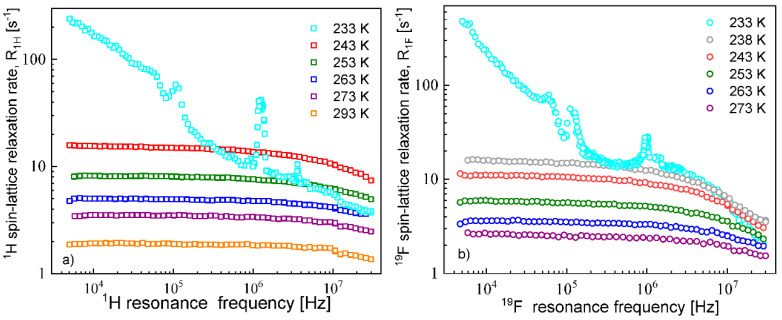
^1^H (**a**) and ^19^F (**b**) spin–lattice relaxation ratesfor 1-ethyl-3-methylimidazolium bis(fluorosulfonyl)imide (EMIM-FSI) in bulk.

The ^1^H and ^19^F spin–lattice relaxation dispersion profiles collected for EMIM-FSI in bulk are shown in Figure 1.

In both cases, for ^1^H and ^19^F, the relaxation data at 238 K and above show a weak dispersion (become dependent on the resonance frequency only in the high frequency range), in contrast to the data at 233 K, that show not only a strong dispersion but also effects referred to as quadrupole relaxation enhancement (QRE) [11,32,33,34,35,36,37,38]. The QRE effects originate from dipole–dipole couplings between ^1^H and ^14^N nuclei in the case of the cation and ^19^F and ^14^N nuclei in the case of the anion. ^14^N nuclei experience quadrupole interactions (because of the spin quantum number *S* = 1, being larger than 1/2) in an electric field gradient. Consequently, when the dynamics is slow (so the quadrupole interaction is not averaged out as a result of the molecular motion) the energy level structure of the ^14^N nuclei results from a superposition of quadrupole and their Zeeman interactions. Thus, at some magnetic fields, the ^1^H (or ^19^F) resonance frequency matches one of the transition frequencies of the ^14^N nuclei. For the spin quantum number *S* = 1, this happens at the following frequencies [34,35,36,37,38]: $\nu_{-}=\frac{3}{4}a_{Q}\left(1-\frac{\eta }{3}\right)$, $\nu_{+}=\frac{3}{4}a_{Q}\left(1+\frac{\eta }{3}\right)$ and $\nu_{0}=\nu_{+}-\nu_{-}=\frac{1}{2}\eta a_{Q}$, where $a_{Q}$ and η denote the amplitude and the asymmetry parameter of the quadrupole coupling, respectively. The amplitude is defined as: $a_{Q}$ = $\frac{e^{2}qQ}{h}$, where *Q* denotes the quadrupole moment of the nucleus, while *q* is the *zz* component of the electric field gradient tensor. At these frequencies, the ^1^H (or ^19^F) magnetization is taken over by the ^14^N nuclei. The fast decay of the magnetization manifests itself as a frequency-specific increase (enhancement) in the ^1^H (or ^19^F) spin–lattice relaxation rates. For ^1^H relaxation, the frequencies at which one clearly sees a relaxation maxima yields: 0.107 MHz, 1.24 MHz and 3.68 MHz. They cannot be associated with a single ^14^N position, because the condition $\nu_{0}=\nu_{+}-\nu_{-}$ is not fulfilled. Analogously, for the ^19^F relaxation, the frequencies take the values: 0.059 MHz, 0.115 MHz and 0.948 MHz, leading to the same conclusion as for the ^1^H relaxation data. We shall come back to this subject in Section 5.

**Figure 2:**
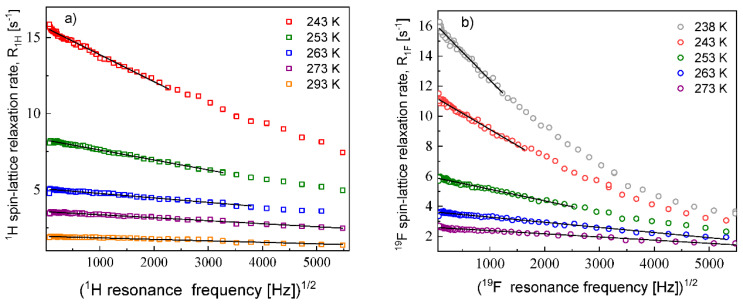
^1^H (**a**) and ^19^F (**b**) spin–lattice relaxation rates for EMIM-FSI in bulk versus the square root of the resonance frequency; lines—linear fits.

The ^1^H and ^19^F relaxation data collected at the higher temperatures allow to determine the relative cation–cation and anion–anion translation diffusion coefficients, respectively. Figure 2 shows the expected linear dependences of the corresponding relaxation rates on the square root of the resonance frequencies in the low frequency range.

The translation diffusion coefficients for the EMIM cations and the FSI anions obtained from Equation (3) are collected in Table 1. The numbers of ^1^H and ^19^F nuclei per unit volume for the EMIM-FSI yield: $N_{H}$ = 3.16 × 10^28^/m^3^, $N_{F}$ = 5.75 × 10^27^/m^3^.

**Figure 3:**
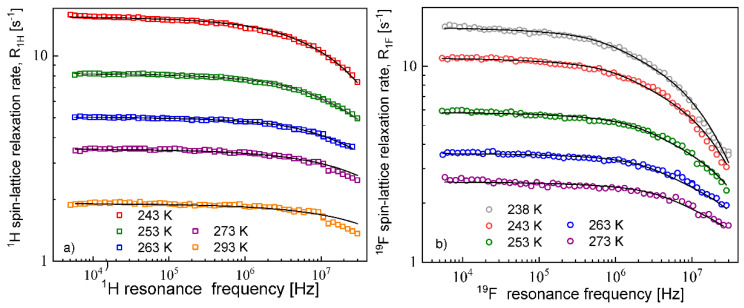
^1^H (**a**) and ^19^F(**b**) spin–lattice relaxation rates for EMIM-FSI in bulk; lines—fits in terms of Equation (7).

The diffusion coefficients were used to reproduce the relaxation dispersion profiles in terms of Equation (7). The results are shown in Figure 3. The fits of the ^1^H spin–lattice relaxation data were given for $d_{H}=d_{cc}$, (the index “*cc*” denotes “cation–cation”): $d_{cc}$ = 2.04 Å (243 K), 2.01 Å (253 K), 1.94 Å (263 K), 2.01 Å (273 K) and 2.08 Å (293 K). An analogous analysis was performed for the ^19^F spin–lattice relaxation data. In this case, we obtained $d_{F}=d_{aa}$ = 1.78 Å (238 K), 1.76 Å (243 K),1.72 Å (253 K), 1.65 Å (263 K) and 1.65 Å (273 K); the index “*aa*” denotes “anion–anion”.

**Figure 4:**
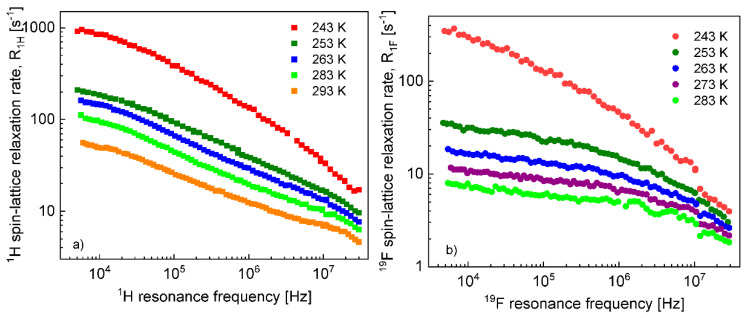
^1^H (**a**) and ^19^F (**b**) spin–lattice relaxation ratesfor EMIM-FSI in the silica matrix.

**Figure 5:**
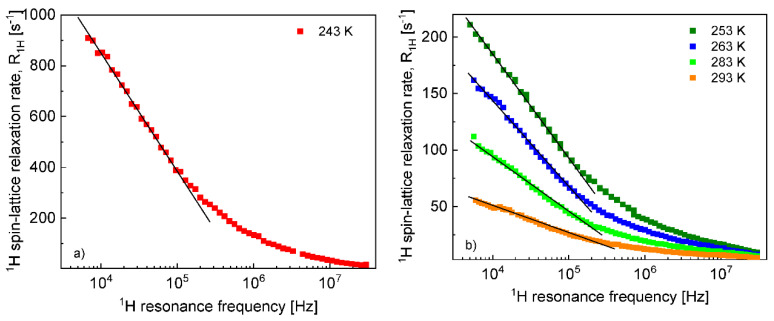
^1^H spin–lattice relaxation rates for EMIM-FSI in the silica matrix at (**a**) 243 K and (**b**) from 253 K to 293 K; lines—linear dependencies characteristic of 2D translation diffusion.

**Figure 6:**
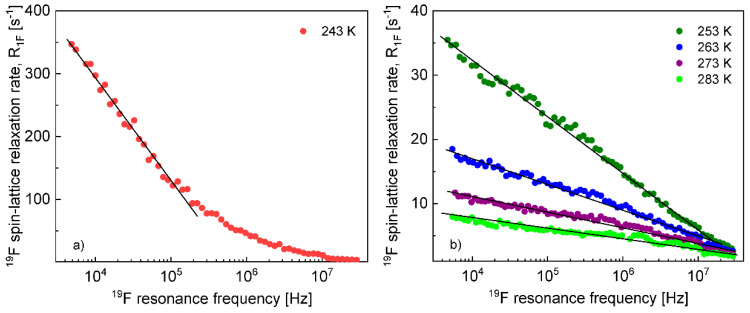
^19^F spin–lattice relaxation rates for EMIM-FSI in the silica matrix at (**a**) 243 K and (**b**) from 253 K to 283 K; lines—linear dependencies characteristic of 2D translation diffusion.

Before proceeding with a quantitative analysis of the relaxation data for the confined EMIM-FSI, it is essential to enquire, in a qualitative way, into the mechanism of the translation diffusion in the confinement. For this purpose, the ^1^H and ^19^F relaxation dispersion profiles for EMIM-FSI in the confinement shown in Figure 4, are displayed in Figure 5 (^1^H) and Figure 6 (^19^F) using the representation $R_{1I}^{conf}\left(\omega_{I}\right)$ versus $ln\left(\omega_{I}\right)$. The linear dependence seen in the low frequency range ($\omega_{I}\tau_{trans}\ll 1$) is a fingerprint of 2D translation diffusion.

**Figure 7:**
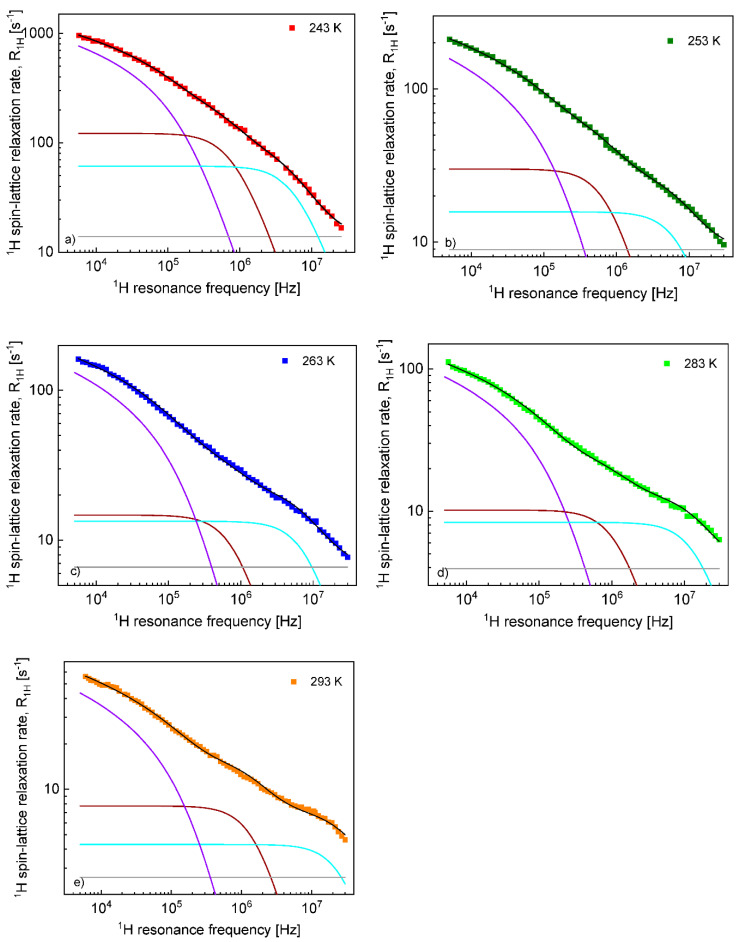
^1^H spin–lattice relaxation data for EMIM-FSI in the confinement at (**a**) 243 K, (**b**) 253 K, (**c**) 263 K, (**d**) 283 K and (**e**) 293 K: $R_{1H}^{conf}$—black line; $R_{1H}^{trans,\;conf}$—violet line; $R_{1H}^{rot,conf,s}$—brown line; $R_{1H}^{rot,conf,f}$—light blue line; and $A_{H}$—grey line.

**Figure 8:**
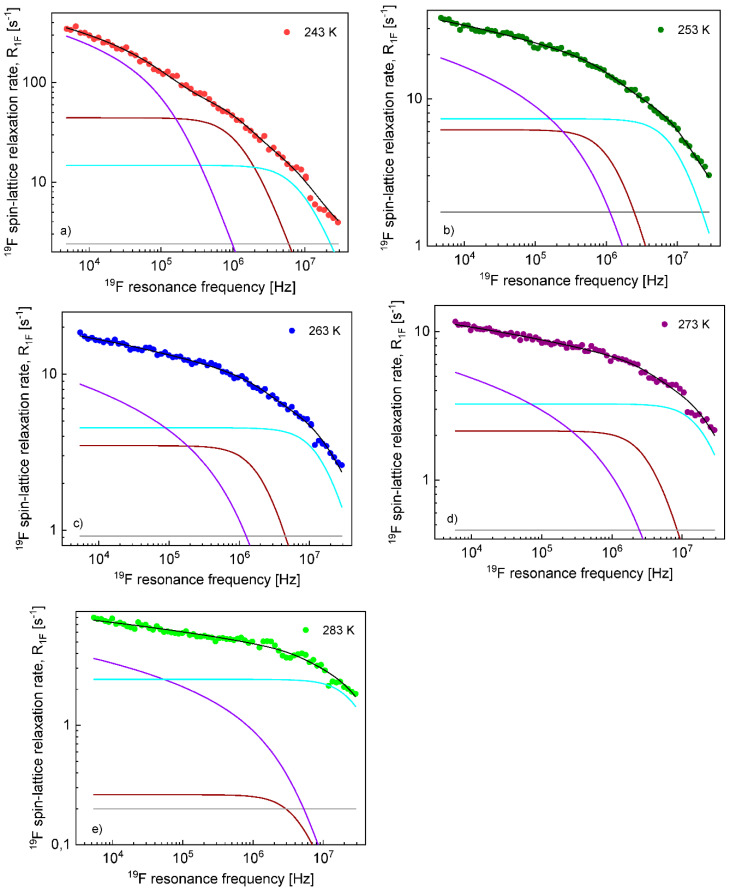
^19^F spin–lattice relaxation data for EMIM-FSI in the confinement (**a**) 243 K, (**b**) 253 K, (**c**) 263 K, (**d**) 273 K and (**e**) 283 K: $R_{1F}^{conf}$—black line; $R_{1F}^{trans,\;conf}$—violet line; $R_{1F}^{rot,conf,s}$—brown line; $R_{1F}^{rot,conf,f}$—light blue line; and $A_{F}$—grey line.

On this basis, the ^1^H and ^19^F spin–lattice relaxation data for EMIM-FSI in the confinement were fitted in terms of Equation (8) with the adjustable parameters: $C_{DD,I}^{trans,conf}$, $\tau_{trans}^{conf}$, $C_{DD,I}^{rot,conf,s}$, $\tau_{rot,I}^{conf,s}$, $C_{DD,I}^{rot,conf,f},\;\tau_{rot,I}^{conf,f},\;A_{I}$. To directly refer tothe cation and the anion dynamics, it is convenient to rename the parameters as $C_{trans\;}^{cation}$, $\tau_{trans,conf}^{cation}$, $C_{DD,conf}^{cation,s}$, $\tau_{rot,conf}^{cation,s}$, $C_{DD,conf}^{cation,f},\;\tau_{rot,conf}^{cation,f},A_{c}$ for ^1^H and $C_{trans\;}^{anion}$, $\tau_{trans,conf}^{anion}$, $C_{DD,conf}^{anion,s}$, $\tau_{rot,conf}^{anion,s}$, $C_{DD,conf}^{anion,f},\;\tau_{rot,conf}^{anion,f},A_{a}$ for ^19^F. The obtained parameters are collected in Table 2, while the fits are shown in Figure 7 and Figure 8 for ^1^H and ^19^F relaxation, respectively. The overall relaxation rates were decomposed into the individual contributions included in Equation (8).

## 5. Discussion

The diffusion coefficients for the EMIM cations in EMIM-FSI obtained by means of NMR relaxometry are in good agreement with those measured by pulsedfield gradient NMR methods obtained from molecular dynamics simulations [39]. To be more specific, the diffusion coefficient reported in [39] at 243 K yields about 9 × 10^−12^ m^2^/s (6.31 × 10^−12^ m^2^/s from NMR relaxometry) and about 6 × 10^−11^ m^2^/s at 293 K (4.36 × 10^−11^ m^2^/s from NMR relaxometry). However, according to our studies, the translation diffusion coefficient of FSI anions is by the factor of about 3, smaller compared to the diffusion coefficient of the cations, while the values reported in [39] are more close. At this stage, one should point out that NMR relaxometry probes relative translation motion. For uncorrelated dynamics, the relative diffusion coefficient is given as a sum of the self-diffusion coefficient (measured by NMR gradient methods) of the interacting species. For identical molecules (ions) the relative diffusion coefficient is twice as large than the self-diffusion coefficient. We applied this relationship to the NMR relaxometry results. The lower values of the self-diffusion coefficient of the FSI anions can indicate a correlated translation motion of the anions. In principle, one can expect that the ^1^H and ^19^F relaxation rates also include contributions associated with the cation–anion, ^1^H-^19^F dipole–dipole interactions mediated by the relative translation diffusion of the ions. Such a contribution is likely more relevant for the ^19^F relaxation, since the number of ^1^H nuclei per unit volume, *N_H_* is larger than the number of the ^19^F nuclei, $N_{F}$. Including the ^1^H-^19^F relaxation contribution would, however, mean more parameters than can hardly be determined, taking into account that the experimental data were reproduced very well without this contribution. Nevertheless, the presence of the ^1^H-^19^F dipole–dipole interactions can, to the same extent, affect the determined diffusion coefficients. Cation–anion interactions manifest themselves by the QRE effects observed at 233 K in the solid-state phase of EMIM-FSI. These effects are associated with the presence of ^14^N nuclei (possessing quadrupole moments). The molecule includes three ^14^N nuclei: two in the EMIM cation and one in the FSI anion. As already pointed out, the positions of the frequency-specific relaxation maxima cannot be explained as a result of ^1^H (^19^F) dipole–dipole coupling with a single ^14^N nuclei. In other words, one needs two sets of the quadrupole parameters ($a_{Q}$ and η) to explain the positions of the relaxation maxima. Due to the structure of EMIM cations, one can expect that the quadrupole parameters for the ^14^N nuclei of EMIM are similar. This implies that the^1^H nuclei of EMIM cations interact not only with their own ^14^N nuclei, but also with the ^14^N nucleus of the TFS anion. Analogously, the ^19^F nuclei of TFS interact with the ^14^N nuclei of TFS and EMIM, at least inthe solid phase. The analysis of the relaxation data at the higher temperatures confirms the 3D character of the translation diffusion of both kinds of ions.

In contrast to EMIM-TFS in bulk, both kinds of ions perform 2D diffusion in the confinement. For EMIM cations, the translational correlation time, $\tau_{trans,conf}^{cation}$, is very weaklydependent on temperature, ranging between 3.71 × 10^−7^ s at 243 K and 3.43 × 10^−7^ s at 293 K. Assuming a similar cation–cation distance of the closest approach for bulk (about 2 Å), one can estimate the diffusion coefficient as being about 5.7 × 10^−14^ m^2^/s, which is about two orders of magnitude lower compared to the value obtained for the bulk liquid at 243K and about three orders of magnitude lower at 293 K. The 2D diffusion can be considered as a sequence of loops near the confining walls interrupted by time periods during which the ions are attached to the surface. Consequently, the correlation time (and hence, the diffusion coefficient) should be treated as an “effective” quantity that reflects both effects. It is not possible to resolve whether the apparent slowing down of the diffusion process indeed stems from a slower movement of the cations near the surfaces or the time scale of the diffusion remains comparable to the diffusion in bulk liquid, but the “residence” life time (time during which the cations are attached to the walls) is long. Taking into account that the values of $\tau_{trans,conf}^{cation}$ are barely dependent on temperature, one may suppose that they rather reflect the “residence” life time, because the time scale of diffusion usually significantly changes with temperature. The values of the dipolar relaxation constant, $C_{trans\;}^{cation}$, decrease with increasing temperature. This effect may suggest that, in fact, there are two fractions of cations in the confinement: a bulk-like fraction that undergoes 3D diffusion (on a time scale similar to that for bulk liquid) in the core of the pores (far from the confining walls) and a fraction near the surface. The population of the last fraction can decrease with increasing temperature and this is reflected by the decreasing dipolar relaxation constant. Comparing the ^1^H spin–lattice relaxation rates for EMIM-FSI in bulk and the confinement, one can easily observe that the relaxation process in bulk is much slower than in the confinement. Consequently, the contribution associated with the bulk-like fraction is likely masked by the other relaxation terms, especially as the number of ^1^H nuclei per unit volume, $N_{H}$, is lower for the bulk-like fraction than in bulk (a part of the cations remains attached to the walls).

The relaxation contribution associated with the intra-cation ^1^H–^1^H dipole–dipole interactions was interpreted in terms of two processes characterized by the parameters: $C_{DD}^{cation,s}$, $\tau_{rot}^{cation,s}$ and $C_{DD}^{cation,f}$, $\tau_{rot}^{cation,f}$.One should explain at this stage that this concept could be replaced by another one—namely a heterogeneous dynamics described by a distribution of correlation times. Typically, the Cole–Davidson function [40,41,42,43] originating from dielectric spectroscopy is used for this purpose. The function includes a phenomenological parameter β (0<β≤1). The parameter isconsidered as a fingerprint of the dynamical heterogeneity of the system; for β=1, the function converges to the Lorentzian form. Sometimes, the concept goes even further and the parameter β is treated as a result of molecular (ionic) interactions in analogy to the mode coupling theory [43] developed for glass-forming liquids. In our opinion, one should not attribute any elaborated meaning to this parameter—it merely reflects, in a phenomenological manner, deviations from the oversimplified model of isotropic molecular (ionic) tumbling. Therefore, instead of pursuing the concept of dynamical heterogeneity, we decided to recourse to two relaxation terms (which one associated with a single correlation time). We could offer two explanations for the existence of the two terms, although we can hardly prove them (one should, however, take into account that the concept of a heterogeneous dynamics cannot be proved, either). One might attribute the terms to the anisotropic rotation of the cations (they are far from being spherical). An alternative explanation could be associated with a restricted dynamics of cations attached to the confining walls. One can invoke here the Lipari–Szabo model [44] for molecules experiencing a local, anisotropic dynamics and undergoing at the same time an overall motion on a much longer time scale. The dynamics can be described by a correlation function that takes the form: $C\left(t\right)=\left(1-S\right)\exp\left(-t/\tau_{f}\right)+S\exp\left(-t/\tau_{s}\right)$, where $\tau_{f}$ and $\tau_{s}$ denote the correlation times of the fast and slow dynamical processes, respectively; S is referred to as an order parameter. This formula means that in the first step (in a short time) the correlation function decays from unity to the S value as a result of the fast, anisotropic motion, and then, in the second step, it eventually decays to zero at long times due to the slow motion. We would not like to speculate with this respect. It is, however, worth noting that the relaxation constant $C_{DD}^{cation,s}$ changes with temperature—it decreases in the temperature range from 243 K to 263 K and then becomes temperature independent. At the same time, the relaxation constant $C_{DD}^{cation,f}$ also decreases with increasing temperature, but it “stabilizes” already at 253 K. This effect might be attributed to the internal dynamics of EMIM cations, especially the dynamics of the chains that include ^1^H nuclei contributing to the dipolar relaxation constants—they can increase at lower temperature as a consequence of a slower and/or more restricted dynamics of the chains. The correlation times $\tau_{rot}^{cation,s}$ and $\tau_{rot}^{cation,f}$ differ approximately by an order of magnitude: $\tau_{rot}^{cation,s}$ varies from 1.06 × 10^−7^ s at 243 K to 4.98 × 10^−8^ s at 293 K, while $\tau_{rot}^{cation,f}$ varies in this temperature range from 1.43 × 10^−8^ s to 2.73 × 10^−9^ s. The frequency independent term, $A_{c}$, can include inseparable contributions, such as a relaxation contribution associated with the dynamics of the cation chains, a contribution originating from the fast rotational dynamics of the bulk-like fraction and/or the relaxation terms originating from ^1^H-^19^F dipole–dipole couplings as hetero-nuclear terms include spectral densities taken at a difference between the resonance frequencies of the participating nuclei [3,4,5,6,7] and because of the ^1^H and ^19^F resonance frequencies are similar, the spectral density does not exhibit a frequency dependence (it is almost like taking a spectral density at zero frequency).

In contrast to the translational correlation time for EMIM cations, the correlation time for TFS anions in the confinement, $\tau_{trans,conf}^{anion}$, considerably changes with temperature: from 4.02 × 10^−7^ s at 243 K to 1.68 × 10^−8^ s at 283 K. Setting the anion–anion distance of the closest approach to 1.7 Å (the averaged value obtained for the liquid in bulk), one can estimate the corresponding translation diffusion coefficient as being approximately 3.6 × 10^−14^ m^2^/s at 243 K and 8.6 × 10^−14^ m^2^/s at 283 K, about two orders of magnitude lower compared to the diffusion coefficients in bulk. The influence of temperature on the correlation time $\tau_{trans,conf}^{anion}$ (and hence, the corresponding diffusion coefficient) is similar to the case of EMIM-FSI in bulk. This suggests that in the case of FSI anions, the $\tau_{trans,conf}^{anion}$ represents the time scale of the diffusion process near the confining walls rather than the “residence” life time. The dipolar relaxation constant, $C_{trans\;}^{anion}$, also decreases with increasing temperature, but it becomes temperature independent at 263K and above. Regarding the rotational dynamics, the correlation times $\tau_{rot}^{anion,s}$ and $\tau_{rot}^{anion,f}$ differ approximately by an order of magnitude, in analogy to the correlation times for EMIM cations. Generally, the rotational correlation times for FSI anions are shorter than for EMIN cations, but the ratio does not exceed factor 3. As far as the dipolar relaxation constants $C_{DD}^{anion,s}$ and $C_{DD}^{anion,f}$ are concerned, the parameters remain temperature independent starting from 253 K; only at 243 K are the values different (larger). In analogy to EMIM cations, the parameters (the rotational correlation times and the corresponding dipolar relaxation constants) may be associated with the anisotropic rotation of the anions. The frequency-independent term, $A_{a}$, may include ^1^H-^19^F relaxation terms (more specifically, the spectral density taken at a difference between the ^1^H and ^19^F resonance frequencies) and/or a relaxation term representing a fast, internal motion of the anions or/and reflect the fast internal motion of the anion.

It is also worth mentioning that a model referred to as “rotation-mediated translation diffusion” (RMTD) has been used for interpreting relaxation NMR relaxation data for confined ionic liquids [45,46]. The model assumes that translational diffusion along rough surfaces leads to rotation—in fact, a reorientation of the dipole–dipole axes being a result of a chain of acts of absorption to a rough surface. This concept allows to explain a low frequency relaxation plateau observed for some systems. The effect is not observed for the data set presented in this work. In reference [47,48], interactions of OH groups at the silica surface have been discussed. One can expect that the interactions lead to the steric hindrance of the rotational dynamics, likely causing a more pronounced anisotropy of the rotational dynamics. Ions near the pore wall surface show slowed dynamics, in contrast to the ions in the core of the pores. Consequently, one can distinguish two fractions of ions of a different mobility.

## 6. Conclusions

^1^H and ^19^F spin–lattice relaxation studies were performed for EMIM-FSI in bulk and mesoporous MCM41 silica matrix confinement in the frequency range from 5 KHz to 30 MHz (referring to ^1^H resonance frequency, and the corresponding range for ^19^F nuclei). The temperature range encompasses 243 K–293 K for ^1^H in bulk, and 243 K–283 K for ^1^H in the confinement with 238 K–273 K for ^19^F in bulk and 243 K–283 K for ^19^F in the confinement. The 3D character of the ionic diffusion in bulk was confirmed by revealing the linear dependencies of the ^1^H and ^19^F spin–lattice relaxation rates on a squared root of the corresponding resonance frequencies. The translation diffusion coefficients of EMIM cations and FSI anions were determined on the basis of the linear dependencies and confirmed by performing a full analysis of the whole relaxation dispersion profiles (relaxation rates versus resonance frequency). As far as the dynamics of EMIM cations and FSI anions in the confinement is concerned, it was shown that both kinds of ions perform 2D diffusion in the vicinity of the confining walls. A thorough analysis of the relaxation dispersion profiles provided values of the correlation times, on the basis of which the corresponding diffusion coefficients were estimated. The correlation times reflect a translation movement mediated by time periods during which the ions stay attached to the confining walls. It was found that for EMIM cations, the correlation times are almost temperature independent, which might suggest that they rather reflect the long “residence” life times than the time-scale of the motion during the diffusion loops. For FSI anions, the correlation time is temperature dependent and ab out two orders of magnitude longer compared to the values obtained for the bulk liquid. Regarding the rotational dynamics, two correlation times (together with the corresponding dipolar relaxation constants) were determined for both kinds of ions, which might be attributed to the anisotropic rotation of the ions. 

## Figures and Tables

**Table 1:** Translation diffusion coefficients of the EMIM and FSI ions in the EMIM-FSI in bulk; the numbers in parentheses indicate the uncertainty of the values; $D_{trans}^{cation}$ denotesthe translation diffusion coefficient of EMIM cations, while $D_{trans}^{anion}$ denotes the translation diffusion coefficient of the FSI anions.

Temperature (K)	$D_{trans}^{cation}$ (m^2^/s)	$D_{trans}^{anion}$ (m^2^/s)
238	-	1.41 × 10^−12^ (2.5%)
243	6.31 × 10^−12^ (1.6%)	2.04 × 10^−12^ (2.0%)
253	1.23 × 10^−11^ (1.2%)	3.97 × 10^−12^ (1.9%)
263	2.09 × 10^−11^ (2.2%)	6.88 × 10^−12^ (1.6%)
273	2.73 × 10^−11^ (1.8%)	9.52 × 10^−12^ (2.3%)
293	4.36 × 10^−11^ (3.1%)	-

**Table 2:** Parameters obtained from the analysis of ^1^H and ^19^F spin–lattice relaxation data forEMIM-FSI in confinement.

**^1^H (Cation Dynamics)**								
**Temp** **(K)**	$C_{trans}^{cation}$ **(Hz^2^)**	$\tau_{trans,conf}^{cation}$ **(s)**	$C_{DD}^{cation,s}$ **(Hz^2^)**	$\tau_{rot}^{cation,s}$ **(s)**	$C_{DD}^{cation,f}$ **(Hz^2^)**	$\tau_{rot}^{cation,f}$ **(s)**	$A_{c}$ **(s^−1^)**	**Relative Error (%)**
243	5.50 × 10^7^	3.71 × 10^−7^	2.30 × 10^8^	1.06 × 10^−7^	8.56 × 10^8^	1.43 × 10^−8^	13.9	3.2
253	1.11 × 10^7^	3.64 × 10^−7^	5.88 × 10^7^	1.02 × 10^−7^	3.15 × 10^8^	1.02 × 10^−8^	8.9	2.9
263	9.50 × 10^6^	3.53 × 10^−7^	3.09 × 10^7^	9.51 × 10^−8^	3.15 × 10^8^	9.50 × 10^−9^	6.6	3.6
283	6.42 × 10^6^	3.47 × 10^−7^	3.09 × 10^7^	6.58 × 10^−8^	3.15 × 10^8^	5.26 × 10^−9^	3.9	3.5
293	3.19 × 10^6^	3.43 × 10^−7^	3.09 × 10^7^	4.98 × 10^−8^	3.15 × 10^8^	2.73 × 10^−9^	2.6	4.6
**^19^F (Anion Dynamics)**								
**Temp** **(K)**	$C_{trans}^{anion}$ **(Hz^2^)**	$\tau_{trans,conf}^{anion}$ **(s)**	$C_{DD}^{anion,s}$ **(Hz^2^)**	$\tau_{rot}^{anion,s}$ **(s)**	$C_{DD}^{anion,f}$ **(Hz^2^)**	$\tau_{rot}^{anion,f}$ **(s)**	$A_{a}$ **(s^−1^)**	**Relative Error (%)**
243	1.90 × 10^7^	4.02 × 10^−7^	1.28 × 10^8^	6.92 × 10^−8^	3.06 × 10^8^	9.63 × 10^−9^	2.4	6.8
253	9.31 × 10^6^	6.28 × 10^−8^	1.99 × 10^7^	6.15 × 10^−8^	1.89 × 10^8^	7.70 × 10^−9^	1.7	4.3
263	3.12 × 10^6^	4.70 × 10^−8^	1.99 × 10^7^	3.49 × 10^−8^	1.89 × 10^8^	4.80 × 10^−9^	0.9	4.7
273	3.12 × 10^6^	2.68 × 10^−8^	1.99 × 10^7^	2.14 × 10^−8^	1.89 × 10^8^	3.45 × 10^−9^	0.5	7.3
283	3.12 × 10^6^	1.68 × 10^−8^	1.99 × 10^7^	1.35 × 10^−8^	1.89 × 10^8^	2.56 × 10^−9^	0.2	10.7
